# Endemic Dengue Associated with the Co-Circulation of Multiple Viral Lineages and Localized Density-Dependent Transmission

**DOI:** 10.1371/journal.ppat.1002064

**Published:** 2011-06-02

**Authors:** Jayna Raghwani, Andrew Rambaut, Edward C. Holmes, Vu Ty Hang, Tran Tinh Hien, Jeremy Farrar, Bridget Wills, Niall J. Lennon, Bruce W. Birren, Matthew R. Henn, Cameron P. Simmons

**Affiliations:** 1 University of Edinburgh, Institute of Evolutionary Biology, Ashworth Laboratories, Edinburgh, United Kingdom; 2 Fogarty International Center, National Institutes of Health, Bethesda, Maryland, United States of America; 3 Center for Infectious Disease Dynamics, Department of Biology, The Pennsylvania State University, University Park, Pennsylvania, United States of America; 4 Oxford University Clinical Research Unit, Hospital for Tropical Diseases, Ho Chi Minh City, Viet Nam; 5 Hospital for Tropical Diseases, Ho Chi Minh City, Viet Nam; 6 Centre for Tropical Medicine, Nuffield Department of Medicine, University of Oxford, Oxford, United Kingdom; 7 Broad Institute of MIT & Harvard, Cambridge, Massachusetts, United States of America; Fundación Instituto Leloir-CONICET, Argentina

## Abstract

Dengue is one of the most important infectious diseases of humans and has spread throughout much of the tropical and subtropical world. Despite this widespread dispersal, the determinants of dengue transmission in endemic populations are not well understood, although essential for virus control. To address this issue we performed a phylogeographic analysis of 751 complete genome sequences of dengue 1 virus (DENV-1) sampled from both rural (Dong Thap) and urban (Ho Chi Minh City) populations in southern Viet Nam during the period 2003–2008. We show that DENV-1 in Viet Nam exhibits strong spatial clustering, with likely importation from Cambodia on multiple occasions. Notably, multiple lineages of DENV-1 co-circulated in Ho Chi Minh City. That these lineages emerged at approximately the same time and dispersed over similar spatial regions suggests that they are of broadly equivalent fitness. We also observed an important relationship between the density of the human host population and the dispersion rate of dengue, such that DENV-1 tends to move from urban to rural populations, and that densely populated regions within Ho Chi Minh City act as major transmission foci. Despite these fluid dynamics, the dispersion rates of DENV-1 are relatively low, particularly in Ho Chi Minh City where the virus moves less than an average of 20 km/year. These low rates suggest a major role for mosquito-mediated dispersal, such that DENV-1 does not need to move great distances to infect a new host when there are abundant susceptibles, and imply that control measures should be directed toward the most densely populated urban environments.

## Author Summary

Although dengue is a major cause of morbidity in many tropical and subtropical regions of the world, little is known about how the causative virus (dengue virus, DENV) spreads through endemic populations. To address this issue we undertook a phylogeny-based analysis of 751 complete genome sequences of DENV-1 sampled from patients in southern Vietnam during 2003–2008. We show that multiple viral lineages co-circulate within the urban area of Ho Chi Minh City (HCMC), and spread at approximately equivalent rates through overlapping geographical areas, suggesting that they are of equivalent fitness. We also observed that DENV-1 within HCMC tended to disperse from more to less densely populated regions, and that this city was the source population for DENV-1 in the rural area of Dong Thap. Despite the high prevalence of DENV-1 in southern Vietnam, viral dispersion rates were relatively low, especially in HCMC where they averaged less then 20 km/year. Such a low rate is consistent with predominantly mosquito-borne spatial dispersal of DENV-1 in this urban setting containing a large number of susceptibles. Together, these results suggest that dengue control measures such as insecticide spraying should be directed toward the most densely populated regions of localities where the virus is endemic.

## Introduction

Dengue is the most important mosquito-borne viral disease of humans, annually responsible for approximately 40 million cases and some 20,000 deaths in tropical and subtropical regions [1]. Dengue is caused by one of four single-stranded positive-sense RNA viruses (DENV-1 to DENV-4, also referred to as serotypes) of the genus *Flavivirus* (family *Flaviviridae*). Despite the large burden of dengue disease, and considerable research effort, there are currently no licensed vaccines or specific therapies. The challenge of effective and safe dengue vaccination is increased by the possibility that imperfect cross-protective vaccination could enhance DENV infection, or even virulence [2], and that lineages within individual DEN viruses, particularly different ‘genotypes’, may also differ in antigenicity [3]–[6]. In addition, the population dynamics of DENV within individual localities are complex, involving the birth-and-death of viral lineages that may also differ in both virulence and fitness [7]–[13], as well as the intricate patterns of gene flow, at both the local and international scales [7], [14], [15].

DENV transmission among humans is largely caused by the urban adapted and anthropophillic *Aedes aegypti* mosquito. Spatial and temporal patterns of dengue prevalence are likely driven by multiple factors including the immune status of human hosts [16], their age [17], [18], virus traits [13], [19], [20], the mosquito vector, and environmental variables including aspects of climate such as levels of precipitation [21], [22]. Human movement must also be an important, but poorly understood, contributor to viral transmission dynamics, and is obviously responsible for the increasingly widespread and complex distribution of the four DEN viruses at the global scale. On a local scale, how much DENV transmission within a specific population is due to the local movement of infected human hosts rather than of mosquitoes is unclear. Understanding the spatial and temporal dynamics of dengue transmission in endemic dengue populations is therefore central to the rational deployment of vector control activities and the design of intervention strategies. In this respect it is critical to determine the spatial structure of DENV within endemic populations, the rate at which DENV lineages diffuse through space (particularly in the face of a partially immune population), whether specific lineages are spreading more rapidly than others and indicative of enhanced fitness, and the likely contribution of mosquitoes and humans to local transmission patterns.

To address these questions we employed a fine-scale molecular approach to characterize the virus population dynamics of a recent DENV-1 outbreak in southern Viet Nam, a region of high dengue endemicity. Between 2006–2008 the estimated incidence of DENV-1 infection in the southern twenty provinces of Viet Nam ranged from 86–190 cases/100,000 [13], markedly higher than during the preceding six-year period when it ranged from 1–28 cases/100,000. The causes of this increased incidence are unknown.

To determine the patterns and dynamics of dengue transmission we utilized an expansive data set of DENV-1 whole genome sequences sampled prior to and during the peak in DENV-1 prevalence over a period of six years (2003–2008). We inferred the dynamics of viral transmission within individual communities, between communities, and between neighboring countries, using recently developed Bayesian phylogenetic methods that utilize both the temporal and spatial information of the sampled sequences. Uniquely, these time-calibrated phylogenetic methods provide the ability to reveal the complex interplay of spatial, genetic and epidemiological dynamics at the local, regional and global scales, and have the ability to consider individual viral lineages, whereas epidemiological approaches based on the analysis of incidence data are at best only able to distinguish among the four DEN viruses.

## Results

### Phylogeography of DENV-1 in South East Asia

We determined the consensus DENV-1 genome sequence (minimum sequence from nt 70–10,400) in acute plasma samples collected from 751 hospitalized patients in urban Ho Chi Minh City (HCMC) (n = 575 sampled between 2003–2008) and rural Dong Thap Province in the Mekong Delta region (n = 176 sampled between 2006–2007). The majority of viruses were sampled from 2006 to 2008 during which DENV-1 was the most prevalent serotype in circulation (Figure S1).

**Figure 1:**
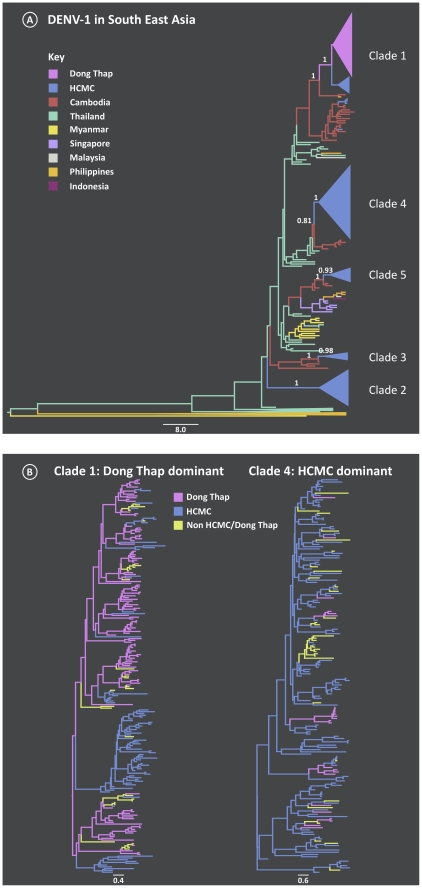
Maximum Clade Credibility (MCC) trees from the discrete phylogeography analysis of DENV-1. A) South East Asia tree reconstructed from the E gene, where the branches are colored by location of viral samples. The number above the branch indicates the posterior probability support for the Vietnamese clades and with nearest sister clades; B) An in-depth look at Vietnamese clades 1 and 4 which were found to be Dong Thap dominant and HCMC dominant, respectively. The branches are colored by sampling location (Dong Thap, HCMC or Non- HCMC and Dong Thap).

To determine the evolutionary relationships of DENV-1 in Viet Nam in the context of surrounding countries we analyzed the envelope (E) gene sequences from these locations (Figure 1A). The 751 DENV-1 sequences sampled from Viet Nam fell into one of five clades within the broader Genotype I cluster of viruses [23]. Four of the five clades consistently clustered within the diversity of Cambodian viruses with good support (posterior probability ranging from 0.81 to 1.0). This phylogeographic evidence, coupled with Cambodia and Viet Nam's shared border, is compatible with Cambodia acting as the major source of Vietnamese DENV-1. A caveat to this is the lack of contemporaneous DENV-1 sequences from nearby Thailand, which has previously been shown to harbor substantial DENV diversity and importation into Viet Nam [13]. Clearly, wider sampling in both time and space is needed to test this hypothesis.

The majority of the clades largely comprised viruses from HCMC, with the exception of clade 1, which was found to be Dong Thap dominant. The timing of these inferred introductions were gauged from the age of the most recent common ancestor (TMRCA) of each clade (Table 1). The period in which these different viral clades emerged in southern Viet Nam ranged from late 2001 to mid-2005. Apart from clade 1, which was found to be the most recent introduction, the mean ages of clades 2–5 did not differ significantly, suggesting that different viral lineages were imported over short or similar time-scales, and then co-circulated. These clades were chosen for more detailed phylogeographic analysis. Finally, genome-wide rates of nucleotide substitution – at ∼1×10^−3^ nucleotide substitutions per site, per year (Table 1) – were the same among clades and highly consistent with those previously determined for DENV [24], [25].

**Table 1:** Rate of nucleotide substitution of DENV-1 for each clade in Viet Nam, and the inferred time of the most recent common ancestor (TMRCA).

		Rate of evolution (10^−3^ subs/site/year)		
Clade	Mean TMRCA	Mean	Lower HPD	Upper HPD
1	2005.22	0.878	0.776	0.980
2	2002.35	0.966	0.864	1.071
3	2003.69	0.949	0.622	1.279
4	2002.94	1.031	0.912	1.155
5	2003.02	0.882	0.656	1.086

### Viral migration between Dong Thap and HCMC

For the clades identified as being within Viet Nam, a discrete spatial model [26] was employed to reveal the migration between the sampling locations. The results are shown in Figure 1B, in which branches are colored by the most probable state location. In four of the five clades HCMC was the most likely viral source, with viruses exported to the rural area of Dong Thap. The non-HCMC isolates in these clades were interspersed among the HCMC sampled isolates, which strongly suggested that the DENV-1 epidemics in southern Viet Nam mainly emerged first in HCMC. The exception was clade 1, which was dominated by Dong Thap viruses and where Dong Thap was inferred to be the most likely place of origin. Moreover, the HCMC viruses in clade 1 did not form a monophyletic group, supporting the view that clade 1 viruses were imported into HCMC on multiple occasions from Dong Thap.

To determine whether the viral migration rates varied between urban and rural epidemics, we compared the spatial dynamics between clades 1 and 4 (Table 2). When focusing on the number of transitions from the inferred source location, a symmetrical pattern was observed between the two clades. For instance, the transmission rate between HCMC and Dong Thap was higher in the HCMC dominant clade 4, while for the reverse direction (Dong Thap to HCMC) it was greater in Dong Thap dominant clade 1. Hence, once a virus became established in a location, rural or urban, the rate of viral exportation was found to be greater than the rate of viral importation.

**Table 2:** Migration between HCMC and Dong Thap (DT) in clade 1 and clade 4, the number of transitions between each state along a branch in the tree using the robust counting method.

Clade 1			
	HCMC	DT	Non DT/HCMC
**HCMC**	-	1.20	5.28
**DT**	12.36	-	11.28
**Non DT/HCMC**	4.73	4.50	-

Clade 4			
	HCMC	DT	Non DT/HCMC
**HCMC**	-	12.60	21.93
**DT**	2.55	-	2.97
**Non DT/HCMC**	4.26	1.83	-

### DENV-1 dispersion in urban and rural locales

The geographical coordinates of the patient's residential address in HCMC (n = 381) or Dong Thap Province (n = 175) was known for 556 cases and this information was employed to reconstruct the fine-scaled dispersion of the individual viral lineages within the sampling areas using a continuous spatial diffusion model with non-homogenous dispersion rates [27]. The average viral dispersion rate (km/year) was calculated for each clade, and separately for HCMC and/or Dong Thap data subsets, as if the epidemic in these regions derived from a single introduction (Table 2). We define virus dispersion rate as a measure of how quickly a virus lineage spreads geographically, given the inferred root location and final sampling locations. Even though we only had one estimate of the average dispersion rate of DENV-1 in Dong Thap, a clear disparity was observed when compared to the rates from HCMC lineages (Table 3). Specifically, the viral lineages from clade 1 in Dong Thap spread approximately 2–3 times faster than any lineage from HCMC. This is indicative of a fundamental difference in the epidemiological dynamics of DENV-1 in the two areas.

**Table 3:** Rate of virus dispersion of DENV-1 and mean age of each clade in the different localities in Viet Nam.

Clade		Mean Dispersion Rate (km/yr)	Lower HPD	Upper HPD	Mean TMRCA
1	Dong Thap	**20.61**	16.72	24.91	2005.42
	HCMC	**11.59**	9.10	13.96	2005.11
	All	**28.68**	25.78	32.12	2005.22
2	HCMC	**7.32**	6.11	8.69	2002.30
	All	**12.44**	10.59	14.56	2002.35
3	All	**38.15**	24.31	52.53	2003.69
4	Dong Thap	**22.18**	10.39	34.56	2003.20
	HCMC	**6.78**	5.66	7.98	2002.43
	All	**18.55**	15.88	21.48	2002.94
5	HCMC	**14.37**	9.43	20.10	2002.33
	All	**23.06**	15.94	29.95	2003.02

**Figure 2:**
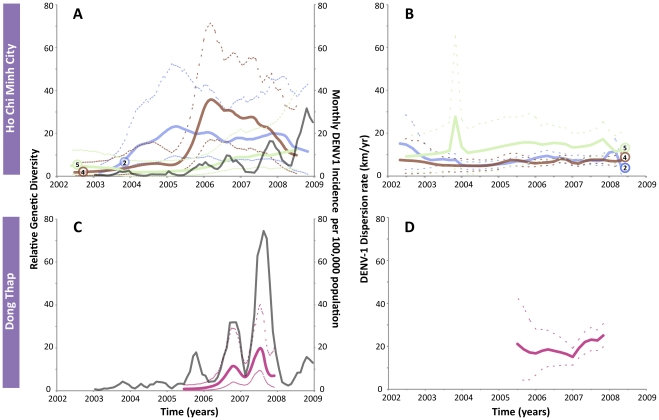
The genetic diversity, dengue incidence rate, and dispersion rates of DENV-1 in HCMC and Dong Thap. The top row shows the results of three dominant lineages in HCMC (clades 2, 4 and 5), while the bottom row shows the results of clade 1 in Dong Thap. Clade 1 is not included in HCMC since it was identified as having multiple origins from Dong Thap. A) Relative genetic diversity of the main clades in HCMC, superimposed with the incidence rate of DENV-1 in HCMC (grey). B) Dispersion rates of the three lineages in HCMC estimated in 6-month intervals from 2003–2009. C) and D) show the results of clade 1 in Dong Thap, relative genetic diversity and incidence rate (grey) and dispersion rate, respectively.

A further dissection of the dispersion rates through time in HCMC (clades 2, 4 and 5) and Dong Thap (clade 1) revealed interesting patterns in the rate of viral spread in the two locations. In HCMC (Figure 2A and B), the monthly incidence of DENV-1 showed a similar trend as in Dong Thap, with corresponding regular fluctuations and an increasing overall trend. However, there was no clear association between genetic diversity, incidence, and dispersion rate observed in the urban environment demonstrated by the roughly horizontal relationship in Figure 2B and the overlapping 95% HPD (highest posterior density) intervals. Hence, although the DENV-1 clades were introduced independently into HCMC, they had spread at similar and effectively constant rates. For Dong Thap, clade 1 was the only one clearly derived from a distinct single importation and of a sufficient size for analysis. The dispersion rate of DENV-1 appeared to be associated with the fluctuations in genetic diversity and monthly incidence in Dong Thap (Figure 2C and D). The two peaks in relative genetic diversity of clade 1 in Dong Thap coincided with the two major peaks in the monthly incidence, indicating that DENV-1 epidemic in Dong Thap is largely driven by this lineage.

**Figure 3:**
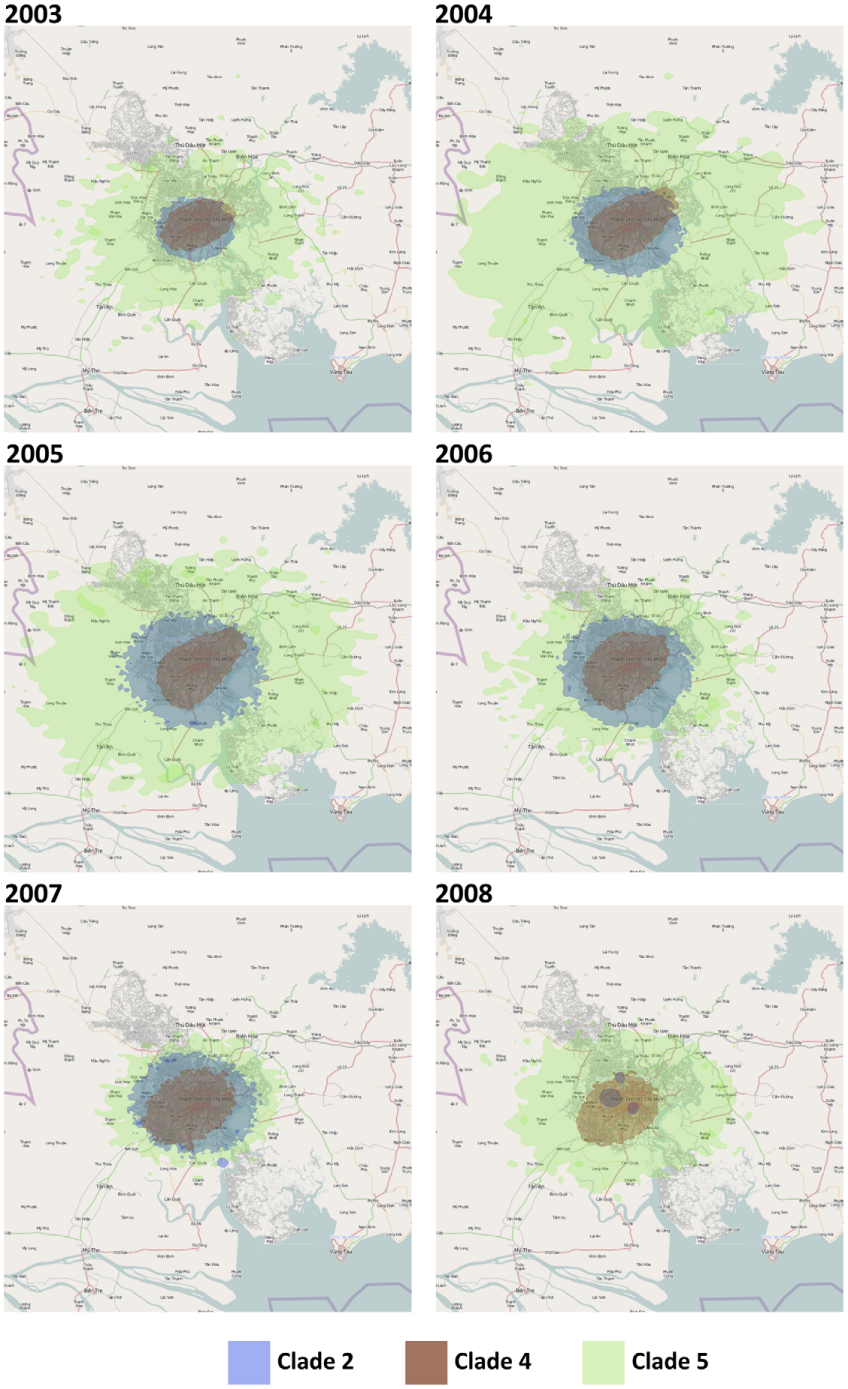
The dispersion of the main clades in HCMC from 2003–2008 estimated from the continuous diffusion phylogeography process. The different clades are color coded (clade 2  =  blue, clade 4  =  red, and clade 5  =  green). All three clades appear to emerge from similar parts of HCMC and continue to co-exist in same geographic space at the same time. Map Data © 2011 OpenStreetMap Contributors, CC-BY-SA.

To investigate whether these dispersion rate estimates in HCMC were simply a reflection of the geographic constraint of our samples, they were re-estimated by randomizing the tip location for each clade (Table 4). The results indicated what the maximum dispersion rate could be given the sampled locations, which were found to be 2–3 times greater than the empirical estimates, with wide HPD intervals (Table 4). The spatial reconstruction of the viral spread at different stages of the epidemics showed that these viral lineages had co-circulated in the same place at the same time (Figure 3). This observation is of fundamental importance as it suggests that the number of susceptible hosts to DENV-1 had not been saturated in HCMC, and could potentially have supported additional DENV-1 lineages in this area.

**Table 4:** Results from randomizing locations at the tips to test the upper limits of the dispersion rates of DENV-1 in HCMC, Viet Nam.

	Empirical			Randomization		
Clade	Mean Dispersion Rate (km/yr)	Lower HPD	Upper HPD	Mean Dispersion Rate (km/yr)	Lower HPD	Upper HPD
HCMC2	**7.32**	6.11	8.69	**21.10**	4.84	155.21
HCMC4	**6.78**	5.66	7.98	**14.32**	4.00	85.69
HCMC5	**14.37**	9.43	20.10	**39.68**	7.84	216.01
joint	**7.49**	6.06	9.31	**20.03**	5.45	92.38

### Population density and transmission routes

**Figure 4:**
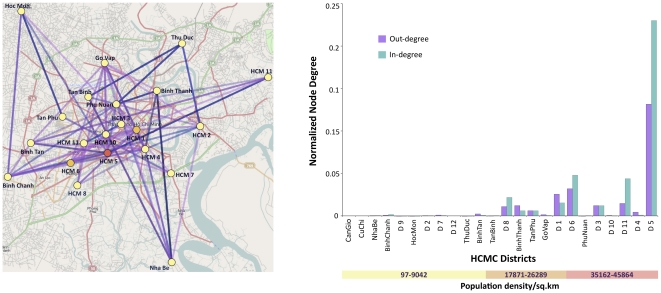
Results from a non-reversible discrete phylogeography analysis of HCMC clades at the district level (21 states). The significant connections at Bayes factor 3 are shown on the map of HCMC. The districts are colored by the number of normalized out-degree connections, where yellow, orange and red indicate low, intermediate, and high, respectively. The number of significant connections to and from a location (district) is represented by the bar chart in terms of in-degree and out-degree respectively. The districts along the x-axis are ordered by increasing population density. Map Data © 2011 OpenStreetMap Contributors, CC-BY-SA.

To determine whether transmission routes within HCMC varied according to population density, we employed a non-reversible discrete phylogeography model applied to district level data. Importantly, the more densely populated inner city districts (above 30,000 people per km^2^) were found to contribute significantly to DENV-1 transmission compared to the suburban districts (Figure 4). Moreover, the most densely populated region, District 5, had the highest number of connections, providing compelling evidence that this area might be a major hub in the city.

## Discussion

At the scale of South-East Asia, the observation that there is a strong clustering by country indicates that there is a far higher level of DENV-1 gene flow within than between countries. Such a phylogeographic pattern is compatible with relatively short transmission distances for DENV as a whole, including that meditated by mosquitoes. This rather limited spatial movement also sits in marked contrast to that observed in respiratory borne pathogens such as influenza, where there is relatively little clustering by place of isolation even on a global scale [28].

Each of the five clades of DENV-1 we identify has a very recent common ancestry, dating only shortly before the appearance of that clade. Given that dengue is endemic in southern Viet Nam, with DENV-1 circulating there for at least 23 years [29], such recent common ancestry suggests that there is a rapid and continual turnover of viral lineages, as has been increasingly described for this and other DEN viruses [8]–[11], [30], [31]. Less clear is whether these instances of lineage turnover are due to fitness differences between the lineages in question, such that natural selection is preferentially able to favor one lineage over another, or whether there is simply a stochastic die-off. That the three major clades we detect in HCMC co-circulate in the same spatial region with overlapping ranges, and possess broadly equivalent levels of relative genetic diversity, suggests that they are of similar fitness and hence that there is little, if any, competition between them. Consistent with this, we did not observe differences in early plasma viremia levels between patients infected with viruses belonging to the different clades (Figure S2). Indeed, we suggest below that HCMC is likely characterized by a large number of susceptible hosts, which would in turn reduce the extent of selective competition between lineages. More generally, these results indicate that although a specific viral serotype may appear to be endemic in a specific geographic region for an extended period, this does not mean that the same viral clades are involved throughout this period.

A striking result from this study is that the ‘virus dispersion rates’ we estimate appear to be very low, and particularly in HCMC where mean rates were universally <20 km/year. Such low rates are especially noteworthy given the rapidity and geographic scale with which DENV-1 re-emerged as the dominant serotype in southern Viet Nam [13]. We therefore interpret these low rates to mean that urban centers like HCMC are characterized by sufficiently high numbers of susceptible hosts such that the virus does not have to move very far to infect a new host. Such a notion is supported by the fact that higher virus dispersion rates are observed in Dong Thap, which is characterized by an approximately ten-fold lower population density (495 persons/km^2^) relative to HCMC (3024 persons/km^2^), although more estimates are clearly needed from this locality. In addition, the highest levels of viral movement were found in and out of the most densely populated region of HCMC (District 5), suggesting that this well-connected locality acts as a focal point for dengue dispersion within the city. Hence, it is not that DENV-1 moves slowly at a spatial scale in HCMC, but rather that it does not have to move far geographically to continue its transmission.

Although our sample of genome sequences is biased toward those from HCMC, our analysis indicates that DENV-1 generally diffuses from HCMC to Dong Thap. Again, this observation is suggestive of a gravity model of viral transmission, in which spatial diffusion occurs over a gradient of population density, and is compatible with our observation that dispersion rates are associated with the numbers of susceptible hosts. A similar gravity-dependent pattern of virus dispersion was recently suggested for DENV-2 in Viet Nam [14], although the use of a strictly reversible phylogeographic model in that case meant that directionality could not be ascertained with certainty. Combined, these studies strongly suggest that the density of the human host population plays a fundamental role in determining the transmission dynamics of endemic dengue.

Typically, adult *A. aegypti* mosquitoes travel short distances of less than ∼100 m during their average life-span of a few weeks [32]–[34]. The very short distances traveled by DENV-1, particularly in HCMC is consistent with mosquitoes, rather than humans, being responsible for the majority of the spatial spread in HCMC, which is again in part a function of the high density of susceptible hosts. A similarly limited movement of dengue has been reported by recent studies that focused on smaller geographic areas, reflecting the restricted spatial range of mosquito vectors, and corroborating the highly focal pattern of DENV transmission observed in HCMC [15], [35]. It is also notable that the geographical range of the three major clades in HCMC changed little from 2003–2008. As such, the full geographic range of these clades is established very early on as the virus is able to spread rapidly through a susceptible host population. Upon the introduction of a new dengue serotype into Iquitos, Peru, it was noted that early-confirmed cases were scattered throughout the city, suggesting a rapid establishment of the virus when entering a completely naïve population [36]. This observation gives added weight to our conclusion that the dispersion rates of DENV-1 in southern Viet Nam are largely a function of the availability of susceptible hosts.

These results have a number of important implications for the future control of dengue. Most generally, that DENV tends to spread relatively slowly on a spatial scale (such that DENV phylogenies exhibit a strong spatial structure both nationally and internationally) suggests that any future vaccine escape or drug resistance mutations would also spread relatively slowly. In addition, that the dispersion rates of DENV appear to largely reflect the density of human host population, including movement from Ho Chi Minh City to Dong Thap, suggests that future control measures, including mosquito spraying, should be directed toward the densest host populations and preferentially to urban over rural areas.

## Methods

### Patient population

The dengue patients from whom DENV whole genome sequences were determined were enrolled in one of two prospective studies at the Hospital for Tropical Diseases in Ho Chi Minh City, Viet Nam or at Dong Thap Hospital, Dong Thap Province, Viet Nam. The median age of these patients was 12 years (interquartile range 7–17 years) and 51% were male. Serological investigations (IgM and IgG capture ELISAs) were performed using paired plasma samples using methods described previously [37]. DENV serotype and viraemia levels were determined using an internally-controlled real-time RT-PCR assay that has been described previously [38].

### Genomic sequencing

Viral genomes were sequenced using the Broad Institute's capillary sequencing (Applied Biosystems) directed amplification viral sequencing pipeline http://www.broadinstitute.org/scientific-community/science/projects/viral-genomics-initiative). This sequencing effort was part of the Broad Institute's Genome Resources in Dengue Consortium (GRID) project. Viral RNA was isolated from diagnostic plasma samples (QIAmp viral RNA mini kit, Qiagen) and the RNA genome reverse transcribed to cDNA with superscript III reverse transcriptase (Invitrogen), random hexamers (Roche) and a specific oligonucleotide targeting the 3′ end of the target genome sequences (nt 10868 to 10890, AGAACCTGTTGATTCAACAGCAC). cDNA was then amplified using a high fidelity DNA polymerase (pfu Ultra II, Stratagene) and a pool of specific primers to produce 14 overlapping amplicons of 1.5 to 2 kb in size for a physical coverage of 2-fold across the target genome (nt 40 to 10649). Amplicons were then sequenced in the forward and reverse direction using primer panels consisting of 96 specific primer pairs, tailed with M13 forward and reverse primer sequence, that produce 500–700 bp amplicons from the target viral genome. Amplicons were then sequenced in the forward and reverse direction using M13 primer. Total coverage delivered post amplification and sequencing was 8-fold. Resulting sequence reads were assembled *de novo* using the Broad Institute's AV454 assembly algorithm (Henn et al. 2011. in review) and a reference-based annotation algorithm.

All whole genome sequences newly determined here have been deposited in GenBank and assigned accession numbers (Table S1).

### Phylogeographic analyses of DENV-1 in Southeast Asia and Viet Nam

A data set of DENV-1 sequences was collated to include isolates from countries in Southeast Asia that were likely linked to Viet Nam via migration. An alignment of the envelope (E) gene (1485 nt) was assembled for the Southeast Asian and Vietnamese isolates (n = 134 and 751, respectively) to include the broadest range of locations. An initial neighbor-joining tree was constructed in PAUP* [39], using a HKY85 nucleotide substitution model with gamma-distributed rates. This allowed us to make an initial identification of the major clades of DENV-1 in Viet Nam. These Vietnamese isolates were then subsampled (n = 101) to explore their phylogeography in context of the South East Asian isolates. Isolation dates for the South East Asia data set were obtained from GenBank annotations and via personal communication. Where specific dates were not available in terms of day and month, a mid-point of the year of isolation was used.

The spatial dynamics of DENV-1 in Southeast Asia were investigated with a discrete diffusion model [26] using Bayesian Monte Carlo Markov Chain (MCMC) method implemented in BEAST [40]. The phylogeography analysis was executed with a codon-structured SDR06 substitution model [41], a relaxed uncorrelated lognormal clock [42] and a Gaussian Markov Random Field (GMRF) coalescent prior [43] over the unknown phylogeny. The discrete diffusion model used the country of isolation of the sampled sequences to reconstruct the ancestral location states of the internal nodes from the posterior time-scaled tree distribution. The MCMC was run for 50 million generations, sampling every 5000^th^ state, and executed multiple times to ensure adequate mixing and stationarity had been achieved.

### Viral transmission between Ho Chi Minh City and Dong Thap province

Major clades of Vietnamese DENV-1 identified from the broad-scale South East Asian analysis were selected for further study to examine the spatial and temporal variation in Viet Nam. In clades with appreciable numbers of sequences from Dong Thap and HCMC, isolates from these locations were analyzed independently to gauge the regional variation in viral transmission patterns. For the fine-scale analysis, a continuous diffusion model based on a lognormal relaxed random walk [27] was employed to model the DENV-1 spatial dynamics in Viet Nam. For each isolate, the specific sample date and location information in terms of the longitude and latitude of the patient's household were used. Isolates that were identical in sample date and location information were down-sampled so as to reduce the potentially biasing effect of over-sampling of epidemiologically-linked cases. The MCMC runs were evaluated as previously described, and the chain lengths ranged from 50 to 100 million generations, and were sampled regularly to yield 10,000 trees from the posterior distribution.

The viral dispersion rates (km/yr) for each data set were calculated across the tree (i.e. total straight-line distance travelled divided by the total time) and biannually to consider the spatial heterogeneity in a time-scaled framework. Plots of relative genetic diversity over time were reconstructed using the GMRF coalescent prior to reveal the association between the genetic diversity of each group in terms of their evolutionary history [43].

Further discrete phylogeography analyses were performed with the robust counting method [44], [45] to determine the extent of viral migration between Dong Thap and HCMC and whether this varied when the lineage originated in a rural or urban area. In this case, the discrete states were represented by either the isolate being sampled from HCMC, Dong-Thap or neither (non-Dong Thap or HCMC).

### Viral migration within Ho Chi Minh City

For the limiting case of a freely mixing (non-spatially structured) epidemic in HCMC, dispersion rates were estimated whilst randomizing the tip locations during the tree proposal in the MCMC, whilst co-estimating the rates for each independent lineage and the joint DENV-1 diffusion rate. To determine the viral transmission network within HCMC, a non-reversible discrete phylogeography model was applied to all the HCMC isolates, using the district of isolation for the discrete states. The analysis was performed and evaluated as described above with the addition of implementing Bayesian Stochastic Search Variable selection (BSSVS) to identify significant transition rates between locations [26]. The transition rates supported by a Bayes factor of at least 3 were examined further by looking at the number of in-degree and out-degree per district. The number of connections was normalized by the number of samples from the source location in order to reduce the bias from under-represented locations in our data set.

### Ethics statement

Patients (or their parents/guardians) gave written informed consent to participate in each of the studies. The study protocols were approved by the Hospital for Tropical Diseases and the Oxford University Tropical Research Ethical Committee.

## Supporting Information

Figure S1 The incidence and isolation of each dengue serotype between 1998 and 2008 in southern Viet Nam.
(TIF)

Figure S2 Levels of viremia observed in patients infected with different clades of DENV-1. All viremia levels were measured within 72 hours of fever onset in patients enrolled into a prospective clinical study at the Hospital for Tropical Diseases in HCMC. There were no significant differences between viremia levels at enrolment between patients infected with different viral clades.
(TIF)

Table S1 The Genbank accession numbers of the whole genome sequences of the DENV-1 viruses sampled in this study.
(XLS)
