# Supplementary figures and images for: Endemic Dengue Associated with the Co-Circulation of Multiple Viral Lineages and Localized Density-Dependent Transmission
Source: PLoS Pathog. 2011 Jun 2;7(6):e1002064. doi: 10.1371/journal.ppat.1002064 (PMC3107208; doi:10.1371/journal.ppat.1002064)

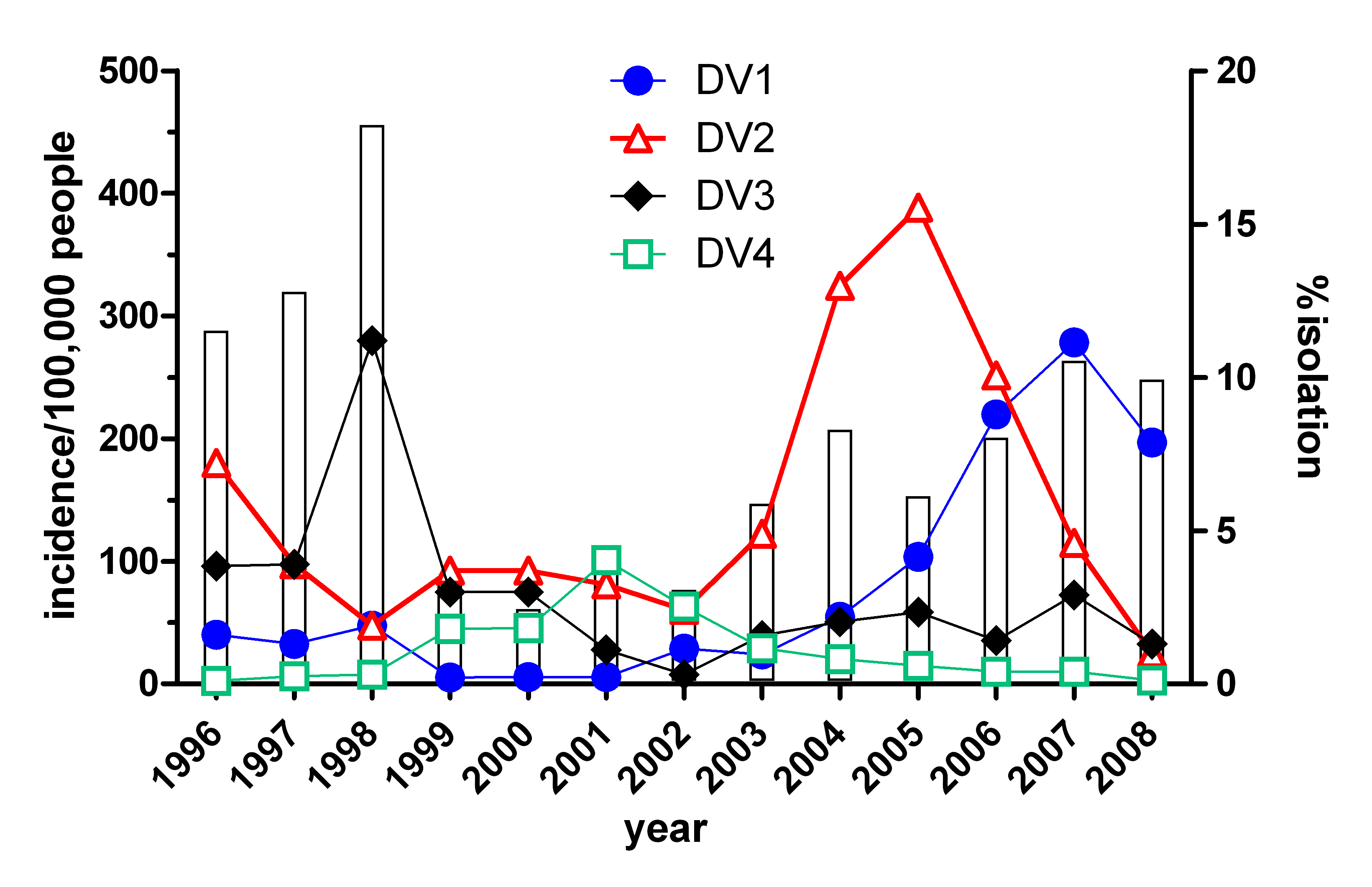

Supplement: Figure S1 — The incidence and isolation of each dengue serotype between 1998 and 2008 in southern Viet Nam. (TIF) [file ppat.1002064.s001.tif]

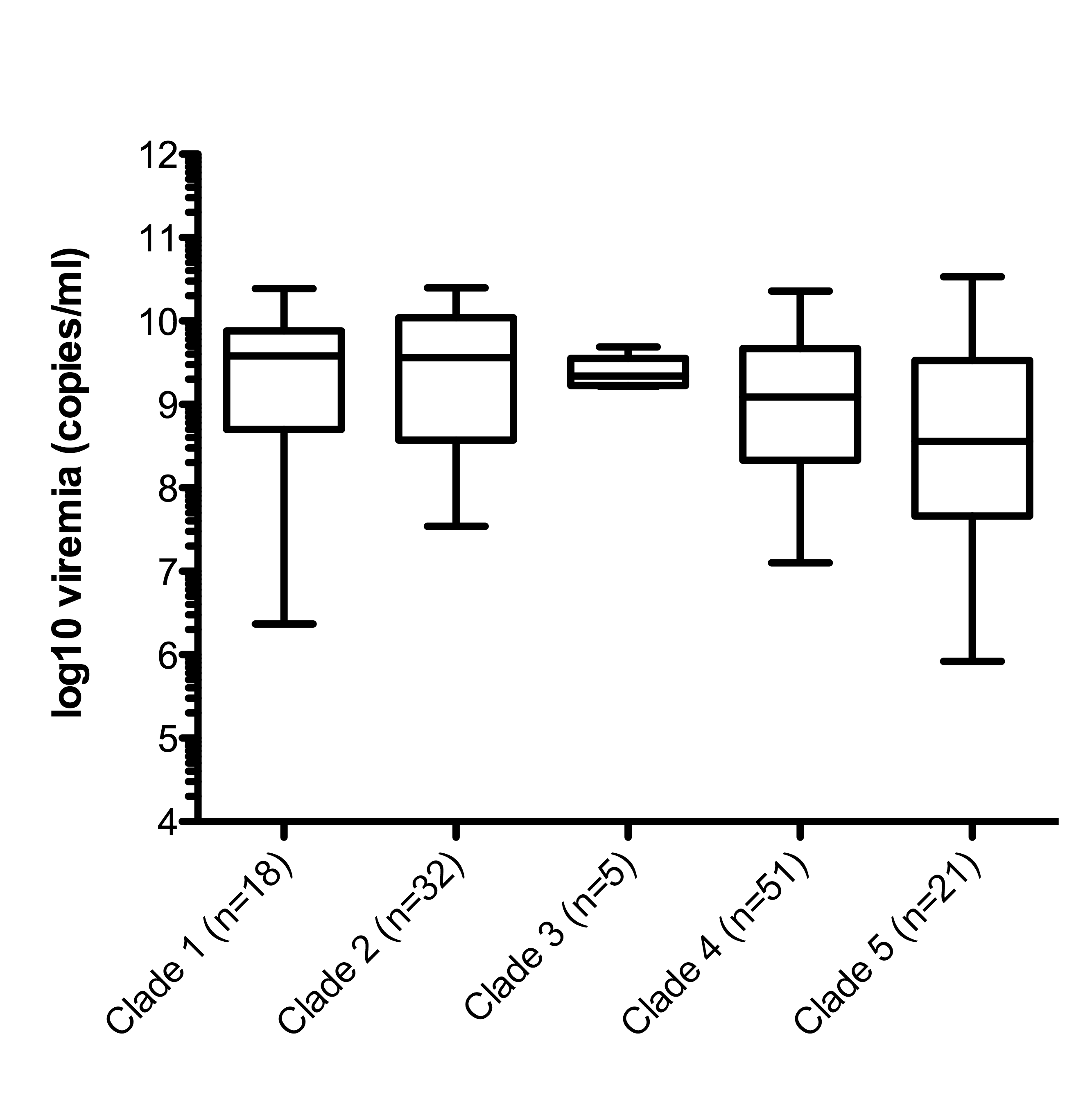

Supplement: Figure S2 — Levels of viremia observed in patients infected with different clades of DENV-1. All viremia levels were measured within 72 hours of fever onset in patients enrolled into a prospective clinical study at the Hospital for Tropical Diseases in HCMC. There were no significant differences between viremia levels at enrolment between patients infected with different viral clades. (TIF) [file ppat.1002064.s002.tif]
